# Testing the efficiency of natural hypovirulence for biological control of chestnut blight under field conditions

**DOI:** 10.3897/imafungus.17.173675

**Published:** 2026-01-26

**Authors:** Simone Prospero, Janine Melanie Schwarz, Marin Ježić, Deborah Marie Leigh, Mirna Ćurković-Perica, Marilena Idžojtić, Zorana Katanić, Ljiljana Krstin, Lucija Nuskern, Ivana Pavičić, Igor Poljak, Kiril Sotirovski, Mihajlo Risteski, Rosemary Vuković, Daniel Rigling

**Affiliations:** 1 Swiss Federal Research Institute WSL, 8903 Birmensdorf, Switzerland Division of Microbiology, Department of Biology, Faculty of Science, University of Zagreb Zagreb Croatia; 2 Agroscope, Agricultural Landscapes and Biodiversity, 8046 Zürich, Switzerland Department of Forest Genetics, Dendrology and Botany, Faculty of Forestry and Wood Technology, University of Zagreb Zagreb Croatia; 3 Division of Microbiology, Department of Biology, Faculty of Science, University of Zagreb, 10000 Zagreb, Croatia Hans Em Faculty of Forest Sciences, Landscape Architecture and Environmental Engineering, Ss Cyril and Methodius University in Skopje Skopje Republic of North Macedonia; 4 Institute of Ecology, Evolution, and Diversity, Faculty of Biosciences, Goethe University Frankfurt, Max-von-Laue-Str. 9, 60438 Frankfurt, Germany Institute of Ecology, Evolution, and Diversity, Faculty of Biosciences, Goethe University Frankfurt Frankfurt Germany; 5 Senckenberg Research Institute, Senckenberganlage 25, 60325 Frankfurt, Germany Swiss Federal Research Institute WSL Birmensdorf Switzerland; 6 Department of Forest Genetics, Dendrology and Botany, Faculty of Forestry and Wood Technology, University of Zagreb, 10000 Zagreb, Croatia Agroscope, Agricultural Landscapes and Biodiversity Zürich Switzerland; 7 Department of Biology, University J. J. Strossmayer of Osijek, 31000 Osijek, Croatia Senckenberg Research Institute Frankfurt Germany; 8 Hans Em Faculty of Forest Sciences, Landscape Architecture and Environmental Engineering, Ss Cyril and Methodius University in Skopje, 1000 Skopje, Republic of North Macedonia Department of Biology, University J. J. Strossmayer of Osijek Osijek Croatia

**Keywords:** *Castanea sativa*, *Cryphonectria parasitica*hypovirus 1, fungus-mycovirus interaction, invasive pathogens

## Abstract

Global increases in connectivity have greatly accelerated the frequency of biological invasions across most of Earth’s ecosystems, including forests. Once invasive organisms become established in a naïve environment, they are difficult to eradicate or contain; thus, management strategies often focus on mitigating their impacts. As the use of chemical pesticides in forests is increasingly prohibited, biological control of pests and diseases has gained importance as an environmentally friendly alternative. Virus-mediated hypovirulence in the chestnut blight fungus *Cryphonectria parasitica* is one of the few successful examples of biological control of an invasive forest pathogen. However, experiments testing the stability of this system *in situ* are still missing. In this study, we conducted a field experiment in chestnut stands with naturally established hypovirulence in Switzerland, Croatia, and North Macedonia to evaluate the effectiveness of CHV1-mediated biocontrol of chestnut blight under different vegetative compatibility (*vc*) type population structures. Our results demonstrate that CHV1 is highly effective as a biological control agent against *C. parasitica*. Artificially initiated bark cankers of various *vc* types were rapidly infected by resident CHV1 strains, which significantly reduced canker growth and sporulation, thereby increasing the survival chances of the infected chestnut sprouts. Under field conditions, vegetative incompatibility barriers proved to be far less restrictive for virus transmission than predicted *in vitro*. Furthermore, our study demonstrates that the immigration of new fungal genotypes into existing cankers is an inherent component of the epidemiology of *C. parasitica*, which significantly contributes to the spread of CHV1. These results are particularly favourable for ensuring the success of hypovirulence-mediated biocontrol of chestnut blight in Europe. However, our conclusions cannot be automatically translated to genetically distant *vc* types from outside Europe, whose accidental introduction should be further avoided.

## Introduction

Global increase in connectivity has significantly accelerated the frequency of biological invasions. Forest ecosystems have not been spared, and the accidental introduction of fungal pathogens or insect pests has resulted in dramatic ecological and economic damage (Ramsfield et al. 2016; Liebhold et al. 2017; Thakur et al. 2019). Once established in a naïve environment, invasive organisms are difficult to eradicate or contain; therefore management measures are often aimed at damage mitigation. Since the application of chemical pesticides in forests is now prohibited in most European countries, control of invasive forest tree pathogens predominantly relies on cultural practices (Prospero and Cleary 2017), resistance breeding (Pike et al. 2021) and/or biological control (Kenis et al. 2019; Prospero et al. 2021).

Virus-mediated hypovirulence in the chestnut blight fungus *Cryphonectria parasitica* (Murr.) Barr is one of the few successful examples of biological control of an invasive forest disease (Ćurković-Perica et al. 2022). *Cryphonectria parasitica* (*Ascomycota*) is a necrotrophic parasite that causes potentially lethal bark lesions (so-called bark cankers) on *Castanea* species (Rigling and Prospero 2018). Introduced in the early 20^th^ century from Asia into North America (Dutech et al. 2012), *C. parasitica* brought the native and highly susceptible American chestnut (*Castanea dentata* (Marsh.) Borkh.) to functional extinction. In Europe, high disease severity was observed on the susceptible European chestnut (*Castanea sativa* Mill.) only in the first two decades after the official detection (1938), after which disease epidemics developed a milder course due to emergence of hypovirulence. Hypovirulence is caused by an infection of *C. parasitica* by the parasitic mycovirus Cryphonectria hypovirus 1 (CHV1); this is a long-established interaction as the virus is present in the native range of the fungus (Hillman and Suzuki 2004). CHV1-infected *C. parasitica* strains are characterised by reduced virulence (Nuss 2005; Rigling et al. 2021) thanks to which they induce superficial or callusing (healing) cankers, which stop expanding (hereafter “passive cankers”), allowing the infected tree to survive (Heiniger and Rigling 1994).

CHV1 has no extracellular phase and can only spread within the fungus. The virus can be transmitted vertically into asexual spores (conidia), but not into sexual ascospores (Anagnostakis 1988; Prospero et al. 2006). Horizontal virus transmission between strains of *C. parasitica* may occur through hyphal anastomosis but is restricted by a vegetative incompatibility system that triggers contact-mediated cell death between incompatible hyphae (Anagnostakis 1977). Vegetative incompatibility in *C. parasitica* is controlled by at least six unlinked diallelic *vic* loci (Cortesi and Milgroom 1998): Two strains are compatible, i.e. belong to the same vegetative compatibility (*vc*) type, if they have the same alleles at all *vic* loci. Virus transmission is restricted between fungal strains which are heteroallelic at *vic* loci, but in nature CHV1 transmission rates between *C. parasitica* strains of different *vc* types have been estimated to be markedly higher than observed under laboratory conditions (Carbone et al. 2004; Bryner et al. 2014). This strongly suggests that the influence of the vegetative incompatibility barrier on CHV1 spread within natural populations of *C. parasitica* should be investigated in more detail to assess the potential for virus transmission, especially in the light of biological control, and ultimately to better understand the host-parasite interactions under field conditions.

Natural hypovirulence is now widespread in many chestnut-growing regions of Europe (Rigling and Prospero 2018), leading to a high survival rate of the infected trees. Nevertheless, the interaction between the European chestnut (*C. sativa*, tree host) – *C. parasitica* (fungal pathogen) – CHV1 (hyperparasite, BCA) is evolutionary young, and its long-term stability is unclear. Environmental conditions (e.g., drought) damaging the host may help the pathogen, resulting in a resurgence of chestnut blight. The introduction of new fungal *vc* types or an increased incidence of rare *vc* types could hinder the spread of CHV1 and reduce the efficiency of hypovirulence-mediated biocontrol.

Previous studies conducted in European chestnut stands with established natural hypovirulence indicated that virus-free *C. parasitica* cankers become virus-infected within a few years and that different *vc* types were detected in the cankers over time (Bissegger et al. 1997; Bryner et al. 2014; Ježić et al. 2018). In these studies, however, fungal strains were distinguished solely by their *vc* type, while CHV1 strains were not further characterized at the molecular level. Additional markers for fungal strains (i.e., microsatellite genotypes) and virus strains (i.e., sequencing) would allow for deeper insights into the infection dynamics in chestnut blight cankers.

In this study, we conducted a field experiment in three different European countries (Switzerland, Croatia, and North Macedonia) where hypovirulence is naturally established to assess the efficiency of CHV1-mediated biocontrol of chestnut blight under different *vc* type population structure. We artificially initiated bark cankers with genetically identifiable virus-free *C. parasitica* genotypes of *vc* types present with varying prevalence in the local fungal populations. In the newly induced cankers, we monitored CHV1 infection incidence, the immigration of new *vc* types and genotypes, and canker development (including growth, activity, and sporulation) over a 26-month period. In addition, CHV1 strains that naturally infected the artificially initiated cankers were partially sequenced for comparison with the resident CHV1 populations and assessed for changes in their diversity over time. This allowed us to test (1) how vegetative incompatibility constraints and CHV1 prevalence in the resident *C. parasitica* population affect the likelihood of canker infection by CHV1, (2) if a CHV1 infection is usually associated with the immigration of new fungal genotypes, and (3) if a CHV1 infection negatively affects canker development, independently from the fungal *vc* type present in the canker.

## Materials and methods

### Study sites and resident *C. parasitica* populations

The study was conducted in six different sites with naturally established hypovirulence located in Switzerland (CH; Contone, Orselina), Croatia (HR; Kašt, Ozalj), and North Macedonia (MK; Kalishte, Smolare), across the distribution range of *Castanea sativa* in central and southeastern Europe (Table 1). All sampled sites consisted of chestnut coppice stands with approximately 15 year-old chestnut sprouts (stem diameter of 6-15 cm). Disease incidence at a specific site (i.e., chestnut sprouts with visible bark cankers) ranged from 62% (Kalishte, MK) to 83% (Smolare, MK) (Table 1). The resident *C. parasitica* and CHV1 populations were sampled in May 2014 before the start of the experiment and characterised: for *C. parasitica vc* type and microsatellite genotype diversity, and for CHV1 subtype and genetic diversity (Ježić et al. 2021). From 25.8% (Smolare, MK) to 75% (Orselina, CH) of the sampled cankers were infected by CHV1 (Table 1) and all viral strains belonged to the Italian subtype (Ježić et al. 2021). In the Swiss and Croatian populations, 8–16 different *vc* types were detected, with EU-1 and EU-2 being the most common ones, whereas all North Macedonian isolates belonged to the *vc* type EU-12 (Ježić et al. 2021).

**Table 1.** Chestnut blight situation at the six experimental sites in Switzerland, Croatia, and North Macedonia and *Cryphonectria parasitica* multilocus genotypes (MLGs) selected for artificial inoculation of healthy *Castanea sativa* sprouts.

	Switzerland (CH)		Croatia (HR)		North Macedonia (MK)	
Characteristics	Contone	Orselina	Kašt	Ozalj	Kalishte	Smolare
Coordinates (N, E)	46.14649, 8.92168	46.18176, 8.79486	45.698015, 15.369578	45.61287, 15.477857	41.138922, 20.643926	41.370943, 22.903064
Altitude (m a.s.l.)	306	647	435	136	789	481
Incidence of chestnut blight^1^	75%	68%	68%	73%	62%	83%
Prevalence of hypovirulence in 2014^2^	57.7%	75%	38.5%	33.3%	46.5%	25.8%
Prevalence of *vc* type in population^3^	EU-1: 16%	EU-1: 29%	EU-1: 42%	EU-1: 28%	EU-12: 100%	EU-12: 100%
	EU-12: 4%	EU-12: 0%	EU-2: 24%	EU-2: 36%		
*C. parasitica* MLGs inoculated (*vc* type)^4^	Cp37 (EU-1)	Cp37 (EU-1)	Cp37 (EU-1)	Cp37 (EU-1)	Cp93 (EU-12)	Cp93 (EU-12)
	Cp21 (EU-12)	Cp21 (EU-12)	Cp2 (EU-2)	Cp2 (EU-2)		

^1^Percentage of infected (i.e., with at least one bark canker) chestnut sprouts. ^2^Percentage of CHV1-infected chestnut blight cankers (Ježić et al. 2021). ^3^Prevalence in the resident *C. parasitica* population of the *vc* types to which the *C. parasitica* MLGs used to initiate the bark cankers belonged. ^4^*Cryphonectria parasitica* MLGs were named as in Prospero and Rigling (2012).

### Artificial inoculations of *Cryphonectria parasitica*

The fungal isolates used for initiating the new bark cankers were selected based on microsatellite markers so that their multilocus genotype (MLG) was not present in the resident *C. parasitica* population (for details see Suppl. material 1). In Switzerland and Croatia, two different MLGs were used for each site: In Switzerland MLGs Cp37 (*vc* type EU-1) and Cp21 (EU-12), and in Croatia MLGs Cp37 (EU-1) and Cp2 (EU-2) (Table 1). Only one MLG (Cp93) of *vc* type EU-12 was inoculated in North Macedonia. The selected MLGs belonged to *vc* types present at different frequencies in the local *C. parasitica* populations, ranging from rare (EU-12 in Switzerland) to common (EU-1 in Switzerland, EU-1 and EU-2 in Croatia) and dominant (EU-12 in North Macedonia) (Table 1). Prior to inoculation, all selected isolates were verified to be virulent (CHV1-free) by culture morphology and RT-PCR as described below.

In August 2014, in Croatia and Switzerland 20 chestnut sprout clusters per site (minimal distance of 5 m to each other) were selected and two healthy sprouts from each cluster were inoculated, each with one of the target *C. parasitica* MLGs. At each North Macedonian site, 25 sprouts from different clusters located at the same distance as in the other two countries were inoculated with the selected MLG. Inoculations were performed as follows. After disinfecting the bark with 70% ethanol, a hole of 5 mm diameter was made to the depth of the cambium using a cork borer. Two mycelial mats punched from the growing edge of cultures from selected *C. parasitica* MLGs (previously grown for seven days at 25 °C in the dark on Potato Dextrose Agar (39 g/L; Difco, Voight Global Distribution, Lawrence, MD)) were placed into the created hole with a spatula. The wound was then sealed with commercially available tree wax to prevent desiccation, as well as bark colonization by other microbes.

### Assessment of canker development

**Figure 1.**
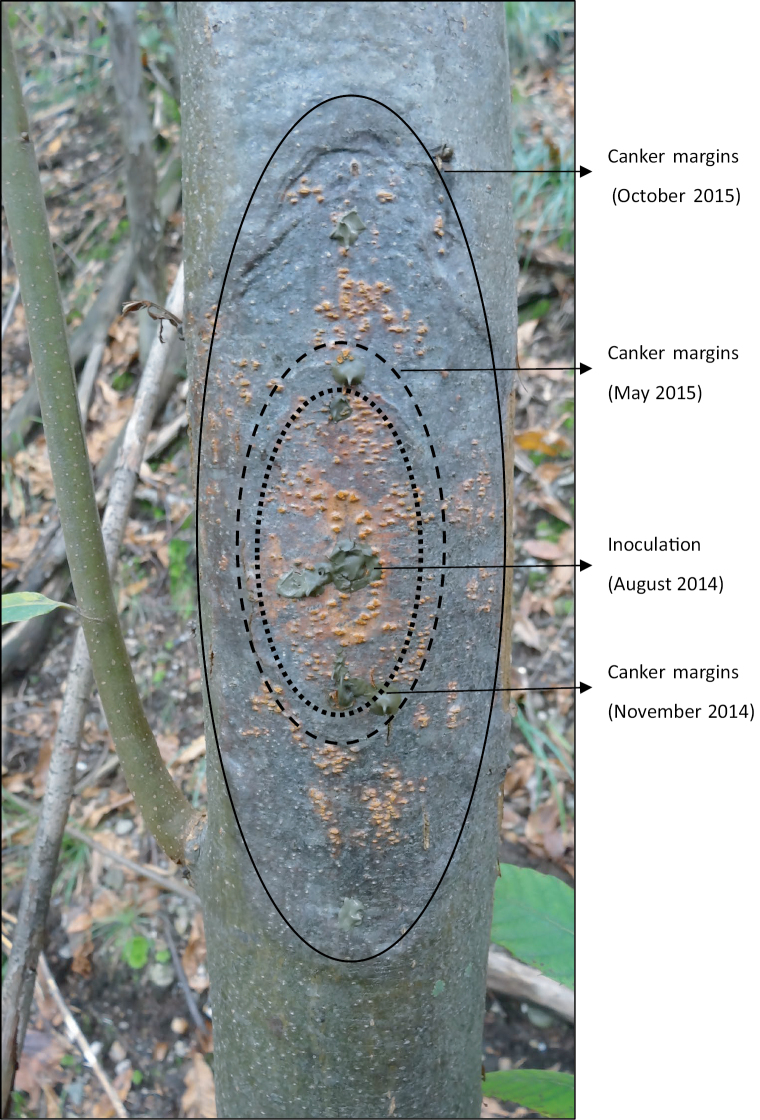
Development over time of an artificially inoculated *Cryphonectria parasitica* bark canker of the *vc* type EU-12 in Contone (Switzerland). After inoculation in August 2014, at each sampling time (November 2014, May 2015, and October 2015) three bark samples (one from the upper margin, one from the middle of the canker, and one from the lower margin) were taken with a biopsy needle (2 mm diameter) and the resulting wounds were covered with tree wax. Note that this specific canker remained virus-free throughout the entire study period.

The development of the artificially initiated cankers was evaluated at four time points over 26 months (November 2014, May 2015, October 2015, and October 2016), by measuring canker length and width with a millimeter ruler, and visually assessing activity (active, with a reddish margin; passive, without a reddish margin, but with a black surface; intermediate) and incidence of stromata (0 = none; 1 = 1–9; 2 = 10–30; 3 > 30 stromata visible) on the canker surface (Bryner et al. 2013; Prospero and Rigling 2016). The area of each canker was calculated for each sampling date using the formula for the surface area of an ellipse. An eventual CHV1-infection or the presence of new *C. parasitica* MLGs in the cankers were determined by re-isolating *C. parasitica* from the artificially inoculated cankers. To this end, three bark samples (upper margin, middle part, lower margin; Fig. 1) were taken from each canker with a bone marrow biopsy needle (Jamshidi gauge, 2 mm diameter; Baxter, Deerfield, IL, USA), which was flame-sterilized between each sampling. The resulting wounds were covered with tree wax. For *C. parasitica* isolation, the bark samples were surface sterilized using 70% ethanol and placed on agar plates. To obtain pure cultures, outgrowing mycelia (ca. 3 × 3 mm) were then transferred to PDA.

To test whether repeated sampling, i.e., artificial canker wounding, would affect CHV1-infection rate (*C. parasitica* is a wound parasite), at the first two dates (November 2014, May 2015) re-isolations were only performed on half of the cankers in each study site.

### Determination of *vc* types and CHV1 infection

The vegetative compatibility (*vc*) types of the *C. parasitica* isolates recovered at each sampling point were determined as follows. First, each isolate was paired on PDA plates with either two (Switzerland and Croatia) or one (North Macedonia) *C. parasitica* isolates used for the inoculations at the specific site, as described by Bissegger et al. (1997). Isolates that were incompatible with the tested *vc* types were further analysed using a multilocus PCR assay (Mlinarec et al. 2018; Cornejo et al. 2019).

CHV1-infected isolates were identified by their specific culture morphology (orange: virus-free; white: virus-infected; Choi and Nuss 1992) on the same PDA plates as for *vc* type testing. CHV1 infection of the white isolates was verified by RT-PCR and sequencing (see below).

### DNA and RNA extraction

*Cryphonectria parasitica* mycelium for RNA or DNA extraction was grown on PDA plates overlaid with cellophane (Celloclaire Inc., Liestal, Switzerland, 80 mm diameter) as described by Hoegger et al. (2000). The mycelia were harvested from the cellophane and DNA and RNA extracted using commercially available extraction kits.

### Microsatellite genotyping of *Cryphonectria parasitica* isolates

All isolates recovered in October 2015 and 2016 from the artificially initiated cankers were genotyped at 10 microsatellite loci (simple sequence repeats, SSR) as described by Prospero and Rigling (2012). PCR products were run on an ABI 3730 sequencer (Applied Biosystems, Carlsbad, CA), using ROX-400 as the internal size standard and allele sizes were scored with the software GeneMapper® 5 (Applied Biosystems™). Multilocus genotypes were named following the convention established in Prospero and Rigling (2012).

### CHV1 sequencing

The purified RNA was transcribed into complementary DNA (cDNA) with random hexamer primers using either the Maxima First Strand cDNA synthesis Kit (Thermo Fisher Scientific) or the GoScriptTM Reverse Transcription System (Promega Corporation, Madison, USA).

Part of the ORF-A region of CHV1 was PCR amplified and sequenced as described by Ježić et al. (2021). Virus sequences were edited with the program CLC Main Workbench 7 (CLC bio). Before assembling forward and reverse sequences, the function *secondary peak calling* was executed with the option *fraction of max. peak height for calling*: 0.8. The assembled sequences were then compared to a CHV1 reference sequence (GenBank Accession Number: JX969928). IUPAC ambiguity codes were used in case of secondary peaks and positions with an uncertain nucleotide. Alignments were separately created for each experimental site and included all sequences recovered from the artificially initiated cankers.

### Statistical analyses

To investigate the effect of a CHV1 infection on the expansion of the artificially initiated bark cankers, the areas of the cankers were compared between virus-infected and virus-free cankers at every sampling date. A canker was termed *virus-infected* for a sampling date, if it yielded at least one virus-infected *C. parasitica* isolate at that date or any date before (i.e., if virus-infected *C. parasitica* isolates were obtained once from a canker, the canker was always considered *virus-infected* afterwards). Areas of virus-infected and virus-free cankers at each date were compared by a one-sided Mann-Whitney U test in the statistical software R.

The effect of canker sampling on virus infection was tested by conducting chi-square tests. For each study site, the frequency of virus-infection in October 2015 was compared between the cankers sampled in November 2014 and May 2015 and the cankers sampled for the first time in October 2015.

### Effect of vegetative incompatibility on virus infection

To assess the effect of vegetative incompatibility barriers on virus infection under field conditions, at each study site we compared the expected CHV1 infection rate for each *vc* type used to initiate the new bark cankers with the observed infection rate of the inoculated cankers for each sampling date. The expected virus infection rates were calculated using the *in vitro* virus transmission rates for each heteroallelic *vic* locus published by Cortesi et al. (2001) and the prevalence of virus-infected isolates in each *vc* type present in the resident populations (data obtained from Ježić et al. 2021). Based on the results of Cortesi et al. (2001), we considered an additive effect for CHV1 transmission rates if more than one *vic* locus was heteroallelic. The expected virus infection rate was then calculated as the mean transmission rate over all *vc* type combinations (*vc* types present in the resident population versus inoculated *vc* types) weighted by the frequency of virus-infected isolates in each population. For North Macedonia, the expected canker infection rate corresponded to the frequency of virus-infected isolates in each population (as published in Ježić et al. 2021), as the inoculated *vc* type was EU-12 and the original populations only consisted of EU-12. The difference between observed and expected virus infection rate was then calculated for each site, *vc* type and sampling date. The correlation between observed and expected infection rates was tested for significance for each sampling date by conducting a Spearman’s correlation test.

### Origin of the CHV1 strains in the cankers

At each site except Smolare (only 15 sequences available), the relationship of the CHV1 strains found in the artificially initiated cankers at the four sampling times (November 2014, May 2015, October 2015, and October 2016) with strains from the resident CHV1 population (May 2014) was investigated by conducting a discriminant analysis of principal components (DAPC, Jombart et al. 2010) with the package *adegenet* (Jombart and Ahmed 2011). For DAPC, all CHV1 strains from the artificial cankers were grouped together. Alignments in FASTA format for every population were read into *R* with the package *ape* (Paradis et al. 2004). After clone-correction (i.e., removal of identical sequences in a population), the dataset consisted of 53 sequences for Contone (24 from the resident population, 29 from the artificial cankers), 58 sequences for Orselina (33/25), 39 sequences for Kašt (10/29), 28 sequences for Ozalj (7/21) and 37 sequences for Kalishte (14/23). Sequences from the Swiss CHV1 populations were 582 bp in length, whereas sequences from the Croatian and North Macedonian populations were 552 bp in length. At each site, genetic differentiation between the resident CHV1 population and the CHV1 population found in the artificially initiated cankers was tested for significance (P < 0.05) by conducting an analysis of molecular variance (AMOVA) in the package *pegas* (Paradis 2010).

### Analysis of CHV1 strains in the cankers over time

For each country, all CHV1 sequences obtained from the cankers at the four sampling dates were collapsed into strains using the online tool FaBox (Villesen 2007). CHV1 strains were defined as those that have identical sequences or differ only by a secondary peak at a single position. The identity and number of strains detected over time in the same cankers were then recorded for each canker from which CHV1 sequences were obtained on at least two sampling dates.

## Results

### CHV1-infection of the cankers

**Figure 2.**
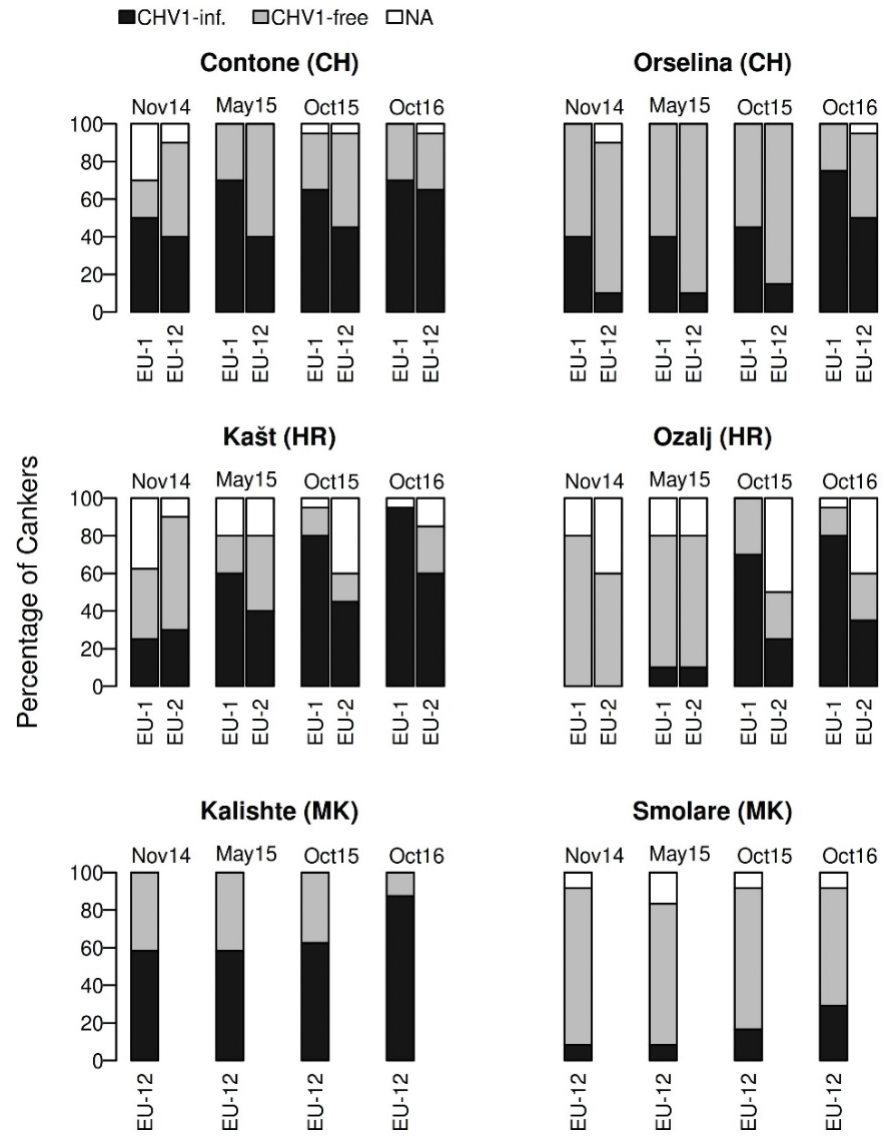
Percentage of CHV1-infected *Cryphonectria parasitica* bark cankers at the four sampling dates (November 2014, May 2015, October 2015, and October 2016) at the investigated sites in Switzerland (Contone, Orselina), Croatia (Kašt, Ozalj) and North Macedonia (Kalishte, Smolare). At the Swiss sites, data is given for the cankers initiated with the two multilocus genotypes of *vc* types EU-1 and EU-12, and at the Croatian sites of *vc* types EU-1 and EU-2; in North Macedonia only EU-12 was used for inoculations. Data in the plots represents cumulative infections, i.e., if a CHV1-infected *C. parasitica* isolate was obtained from a canker once, the canker was always referred to as infected afterwards. CHV1-inf., % cankers infected by CHV1; CHV1-free, % cankers without CHV1; NA, Missing data because of unsuccessful isolation.

At the first sampling in November 2014 (i.e., three months after artificial canker initiation), virus-infected isolates of *C. parasitica* were already recovered from the initially virus-free bark cankers at all sites, except Ozalj (HR), with an incidence ranging from 9.1% (Smolare, MK) to 58.3% (Kalishte, MK) (Fig. 2). At the two Swiss sites, the incidence of CHV1 was higher in cankers of the common *vc* type (EU-1) than in those of the rare *vc* type (EU-12). In Croatia, similar virus-infection rates in cankers of both *vc* types were detected in Kašt (HR) (Fig. 2). In Ozalj, the first virus-infections were observed in May 2015, when 12.5% of the cankers yielded CHV1-infected *C. parasitica* isolates. The overall incidence of virus-infected cankers increased over time at all six sites (Fig. 2). By the end of the experiment (October 2016), 64.1% (Orselina) and 69.2% (Contone) of the cankers at the Swiss study sites were virus infected. The incidence of virus-infected cankers in Croatia was 74.2% (Ozalj) and 86.1% (Kašt), and in North Macedonia 31.8% (Smolare) and 87.5% (Kalishte).

In November 2014 and May 2015 only half of the artificially initiated bark cankers were sampled, while all were sampled in October 2015. This allowed assessment of the effect of sampling (i.e., canker wounding) on virus infection rate. Chi-square tests showed no significant difference (P > 0.05) in virus infection rate between previously sampled and unsampled cankers at all study sites indicating that the sampling procedure applied had no influence on the likelihood a canker became infected with CHV1.

**Figure 3.**
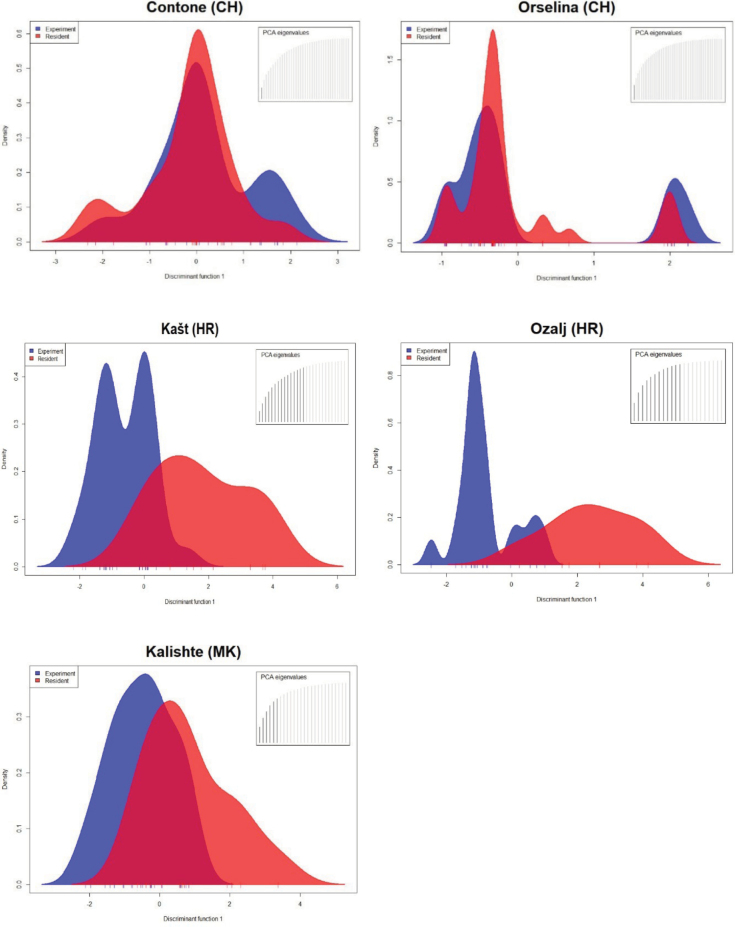
Discriminant analysis of principal components of the CHV1 sequences obtained during the experiment at Contone and Orselina (Switzerland), Kašt and Ozalj (Croatia) and Kalishte (North Macedonia) from the resident canker population (Resident, red) and from the artificially initiated cankers (Experiment, blue).

Based on sequence analysis, 66.2% of the overall artificially initiated cankers that became CHV1-infected during the experiment were infected by a single CHV1 strain which persisted over time (Table 2, for details see Suppl. material 2). This applies to cankers of all *vc* types except EU-1 cankers in Croatia, in which two CHV1 strains were predominant (46.7%). In five CHV1-infected cankers (one EU-12 in Switzerland, three EU-1 in Croatia and one EU-12 in North Macedonia), three CHV1-strains were detected (Table 2). DAPC revealed, at each site, a partial to almost complete overlap of the CHV1 strains from the natural cankers (resident population) and the strains recovered from the artificially initiated cankers (Fig. 3). This was confirmed by AMOVA which revealed no significant differentiation between the two CHV1 populations at any of the sites, with only 0 to 6.8% of the genetic variance explained by differences between the two populations (data not shown).

**Table 2.** CHV1 strains detected in individual *Cryphonectria parasitica* bark cankers on at least two sampling dates.

				Number of CHV1 strains per canker		
Country, inoculated *vc* type	Cankers (No)^1^	CHV1 sequences (No)^2^	CHV1 strains (No)^3^	One	Two	Three
Switzerland						
EU-1	19	102	18	17 (89.5)^4^	2 (10.5)	0 (0)
EU-12	10	57	14	6 (60.0)	3 (30.0)	1 (10.0)
Croatia						
EU-1	15	61	25	5 (33.3)	7 (46.7)	3 (20.0)
EU-2	9	23	10	7 (77.8)	2 (22.2)	0 (0)
North Macedonia						
EU-12	12	52	17	8 (66.7)	3 (25.0)	1 (8.3)
Total	65	295	NA^5^	43 (66.2)	17 (26.1)	5 (7.7)

^1^Number of CHV1-infected cankers from which CHV1 sequences were obtained on at least two sampling dates (November 2014, May 2015, October 2015, October 2016; for details see Suppl. material 2). Cankers from which CHV1 sequences were only obtained on one sampling date are excluded. ^2^Total number of CHV1 sequences analysed. ^3^Total number of CHV1 strains. Strains have an identical sequence or differ by only one secondary peak at a single position. ^4^Percentages in brackets. ^5^Not applicable because CHV1 strains were assigned per country.

### Effect of vegetative incompatibility on CHV1 infection of the cankers

**Figure 4.**
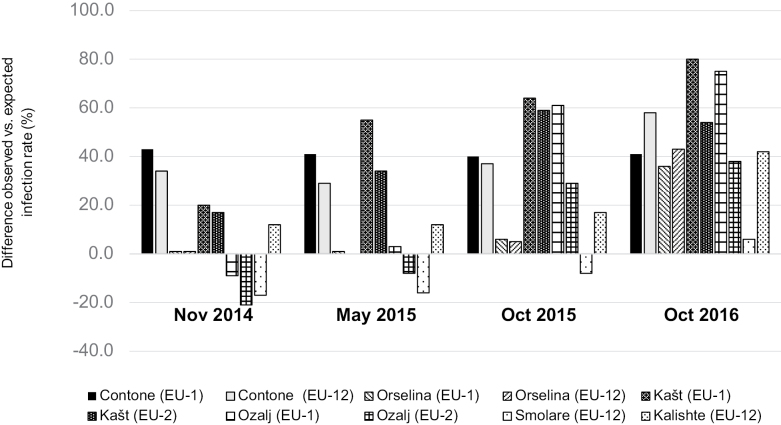
Differences between observed and expected (based on the *in vitro* virus transmission rates for each *vic* locus published by Cortesi et al. (2001)) infection rates with CHV1 of the artificially initiated *Cryphonectria parasitica* cankers in Switzerland (Contone, Orselina), Croatia (Kašt, Ozalj) and North Macedonia (Kalishte, Smolare) at the four sampling dates (November 2014, May 2015, October 2015, October 2016). For details see Suppl. material 1.

From the first (November 2014) to the last (October 2016) sampling at Contone (CH) and Kašt (HR), the observed virus infection rates of the artificially initiated cankers of both *vc* types were higher than those expected based on the *vc* type diversity and CHV1-incidence in the local *C. parasitica* populations (Fig. 4, for details see Suppl. material 3). The other Swiss (Orselina) and Croatian (Ozalj) sites showed initially similar (Orselina) or lower (Ozalj) than expected virus infection rates for cankers of both *vc* types. However, over time CHV1-incidence in the cankers increased and by the end of the experiment it was higher than expected in these two populations as well. In North Macedonia, the two sites showed a similar trajectory of the virus infection rates, but a different initial situation. In Kalishte, the observed infection rate was higher than the expected from the beginning, whereas in Smolare it remained lower than expected until the last sampling (October 2016) at which point it became slightly higher.

Across all sites and *vc* types at the first two sampling dates, a weak to moderate non-significant correlation was present between observed and expected virus infection rates (Spearman’s correlation coefficient: *r_s_* = 0.51, P = 0.13 in November 2014; *r_s_* = 0.38, P = 0.27 in May 2015), which became very weak in the following samplings (*r_s_* = -0.09, P = 0.80 in October 2015; *r_s_* = 0.16, P = 0.65 in October 2016).

### Effect of a CHV1 infection on canker development

**Figure 5.**
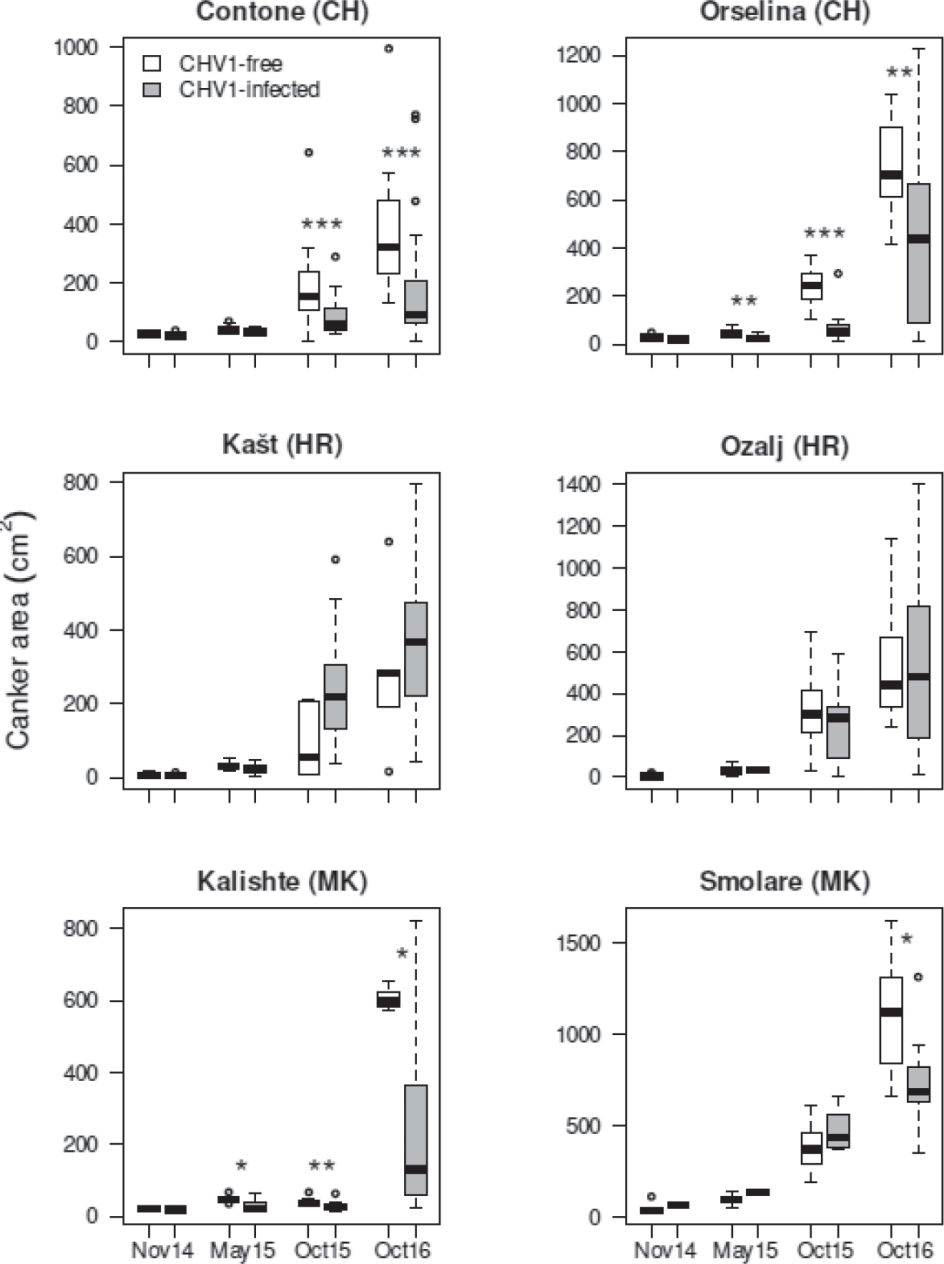
Development of CHV1-free and CHV1-infected *Cryphonectria parasitica* bark cankers during the experiment (November 2014 to October 2016) at the six sites investigated in Switzerland (Contone, Orselina), Croatia (Kašt, Ozalj) and North Macedonia (Kalishte, Smolare). White bars represent non-infected cankers and grey bars CHV1-infected cankers. Significant differences in canker area between CHV1-infected and non-infected cankers at a given time point are marked with asterisks (Mann-Whitney U-test: *, P < 0.05; **, P < 0.01; ***, P < 0.001).

A CHV1 infection had a significant negative effect on canker development in Switzerland and North Macedonia, but not in Croatia (Fig. 5). In Switzerland, significant differences in canker size between virus-infected and virus-free cankers were first observed on the third sampling date (October 2015, i.e. 14 months after inoculation) and became even more pronounced one year later (October 2016). At the two North Macedonian sites, reaction time of canker expansion to a CHV1-infection differed markedly. In Kalishte, size reduction in virus-infected cankers was already significant in May 2015, whereas in Smolare differences were noticeable only at the last sampling in October 2016 (Fig. 5).

Assessment of canker activity at the end of the experiment showed that 42.4% of the overall bark cankers were active (i.e., with a reddish margin suggesting that the fungus is still growing), 33.8% passive (i.e., without a reddish margin, indicating that the canker has stopped expanding) and 23.8% intermediate. Passive cankers were present at all sites with an incidence ranging from 9.1% (Smolare, MK) to 44.4% (Kašt, HR) and were more frequent among virus-infected cankers (39.6%) than virus-free cankers (8.8%).

At the last sampling, 51.3% (Contone, CH) to 100% (Smolare, MK) of the bark cankers had *C. parasitica* stromata on their surface, with a mean estimated value ranging from 1.1 (Contone, CH) to 2.5 (Smolare, MK) out of a maximal possible value of 3. In four out of the six sites more sporulating virus-free than virus-infected cankers were observed, except in Kašt where stromata were observed on 60% of the virus-free and 87.1% of the virus-infected cankers, and Smolare where all cankers had visible stromata. At all sites, the mean estimated value of stromata was higher on virus-free cankers (range: 2–3) than on virus-infected cankers (range: 0.7–2).

### Dynamic of *Cryphonectria parasitica* MLGs within cankers

At all sites for both sampling dates (October 2015 and October 2016), the originally inoculated *C. parasitica* MLGs were recovered from the inoculated bark cankers. However, the overall prevalence decreased to 67.2% of the isolates in October 2015 and further dropped to 56% in October 2016 (Table 3). In Switzerland, the original fungal MLGs were more frequent in isolates from the cankers of the rare *vc* type (EU-12) than of the common *vc* type (EU-1). Cankers of the common *vc* type (EU-1) were more frequently colonized by new *C. parasitica* MLGs of the same *vc* type than by new MLGs of different *vc* types. In Croatia, isolates of the original MLGs and *vc* types were predominant in October 2015 and October 2016 in cankers of both *vc* types. In North Macedonia, at both sampling dates all new MLGs were of the original *vc* type EU-12, which is the dominant *vc* type in local populations.

**Table 3.** Multilocus genotypes (MLGs) and *vc* types of the *Cryphonectria parasitica* isolates recovered in October 2015 and October 2016 from the artificially initiated bark cankers in Switzerland, Croatia, and North Macedonia.

October 2015					October 2016			
Country, inoculated *vc* type	Isolates (No)	Original MLG and *vc* type^1^	New MLG, original *vc* type^2^	New MLG, new *vc* type^3^	Isolates (No)	Original MLG and *vc* type^1^	New MLG, original *vc* type^2^	New MLG, new *vc* type^3^
Switzerland								
EU-1	97	53 (54.6)	38 (39.2)	6 (6.2)	87	41 (47.1)	34 (39.1)	12 (13.8)
EU-12	113	108 (95.6)	0	5 (4.4)	79	70 (88.6)	2 (2.5)	7 (8.9)
Croatia								
EU-1	80	63 (78.8)	16 (20.0)	1 (1.2)	79	49 (62.0)	30 (38.0)	0
EU-2	31	19 (61.3)	5 (16.1)	7 (22.6)	34	22 (64.7)	9 (26.5)	3 (8.8)
North Macedonia								
EU-12	100	40 (40.0)	60 (60.0)	0	85	22 (25.9)	63 (74.1)	0
Total	421	283 (67.2)	119 (28.3)	19 (4.5)	364	204 (56.0)	138 (37.9)	22 (6.1)

^1^Number (in brackets %) of isolates with the same MLG and *vc* type as originally inoculated. ^2^Number (in brackets %) of isolates with a different MLG, but the same *vc* type as originally inoculated. ^3^Number (in brackets %) of isolates with different MLG and *vc* type than originally inoculated.

By the end of the experiment (October 2016), *C. parasitica* MLGs different to the one originally inoculated were found in virus-free and virus-infected bark cankers at a roughly similar proportion (Table 4); about 45% of the cankers had only the original MLG, 45% had mixed MLGs and 10% had only new MLGs. Most of the new MLGs (71.4% in virus-infected cankers and 80.6% in virus-free cankers) belonged to the same *vc* type as the strain initially inoculated to initiate the cankers. In Switzerland, cankers of the rare *vc* type (EU-12) showed a higher prevalence of the original MLG than cankers of the common *vc* type EU-1. This was particularly noticeable for those that were virus-free. CHV1-infected cankers in Croatia showed a similar MLG composition (i.e., original, mixed, new) for both *vc* types originally inoculated. This was not the case for CHV1-free cankers, but this result may be at least partially influenced by the low number of EU-1 cankers (3) from which *C. parasitica* could be successfully isolated. In North Macedonia, 66.7% (CHV1-infected cankers) and 77.8% (CHV1-free cankers) of the cankers yielded mixed MLGs (Table 4).

**Table 4.** Multilocus genotypes (MLGs) of *Cryphonectria parasitica* recovered from the artificially initiated bark cankers in Switzerland, Croatia, and North Macedonia that became CHV1-infected or remained CHV1-free by October 2016. The table shows the combined results of the sampling in 2015 and 2016.

CHV1-infected cankers							CHV1-free cankers					
	No^1^	Original MLG only^2^	Mixed MLGs^3^	New MLGs only^4^	*vc* type of new MLGs^5^		No	Original MLG only	Mixed MLGs	New MLGs only	*vc* type of new MLGs	
					As inoculated	Different					As inoculated	Different
Switzerland												
EU-1	29	9 (31.0)	14 (48.3)	6 (20.7)	10 (50.0)	10 (50.0)	11	2 (18.2)	4 (36.4)	5 (45.4)	7 (77.8)	2 (22.2)
EU-12	23	14 (60.9)	9 (39.1)	0	2 (22.2)	7 (77.8)	15	14 (93.3)	1 (6.7)	0	0	1 (100.0)
Croatia												
EU-1	35	17 (48.6)	13 (37.1)	5 (14.3)	17 (94.4)	1 (5.6)	3	1 (33.3)	2 (66.7)	0	2 (100.0)	0
EU-2	19	9 (47.4)	8 (42.1)	2 (10.5)	6 (60.0)	4 (40.0)	10	5 (50.0)	2 (20.0)	3 (30.0)	2 (40.0)	3 (60.0)
North Macedonia												
EU-12	27	7 (25.9)	18 (66.7)	2 (7.4)	20 (100.0)	0	18	4 (22.2)	14 (77.8)	0	14 (100.0)	0
Total	133	56 (42.1)	62 (46.6)	15 (11.3)	55 (71.4)	22 (28.6)	57	26 (45.6)	23 (40.4)	8 (14.0)	25 (80.6)	6 (19.4)

^1^Total number of cankers, excluding those from which no *C. parasitica* isolates could be recovered in 2015 and 2016. ^2^Number (in brackets %) of cankers from which only the originally inoculated *C. parasitica*MLG was re-isolated in 2015 and 2016. ^3^Number (in brackets %) of cankers from which the originally inoculated as well as new *C. parasitica* MLGs were re-isolated at least in one sampling year. ^4^Number (in brackets %) of cankers from which only new *C. parasitica* MLGs were re-isolated in 2015 and 2016. ^5^Number (in brackets %) of cankers from which new MLGs with inoculated or different *vc* type were recovered. If a canker yielded new MLGs than inoculated and different *vc* types, it was counted as a canker with a different *vc* type.

## Discussion

A great advantage of biological control is its potential sustainability, which eliminates the need for a continuous re-introduction of the BCA (Kenis et al. 2019). However, when the BCA is a parasitic mycovirus occurring exclusively in the cytoplasm of a fungal pathogen, vegetative incompatibility is an important exclusion mechanism that may limit its dissemination in the fungal population. Vegetative incompatibility limits cytoplasmic exchanges between incompatible fungal strains and thus mycovirus spread (Caten 1972). In this study, we tested the efficiency of CHV1, the natural BCA against chestnut blight, in European *C. parasitica* populations with naturally occurring hypovirulence. For this, we artificially initiated bark cankers with virus-free isolates of *C. parasitica* of different multilocus genotypes and *vc* types and followed their development over 26 months (August 2014–October 2016).

Bark cankers artificially initiated with locally occurring *vc* types became infected by CHV1 over the course of the experiment at all experimental sites. Independently of the incidence of the specific canker-causing *vc* type in the local *C. parasitica* population, CHV1 infection probability of the cankers significantly increased with time. Twenty-six months after initiation, 32–88% (depending on the site) of the cankers were infected by CHV1 and at all sites infection rates were higher than expected when using a model based on *in vitro* transmission rates published by Cortesi et al. (2001). These results most likely rely on several conditions: First, the presence of a sufficient amount of hypovirulent inoculum in the local *C. parasitica* population. Since the artificially initiated cankers were infected by resident CHV1 strains, we can assume that the CHV1-infected conidia are produced locally within the study plot or in the surrounding chestnut stands. Given the low ability of *C. parasitica* to sporulate on virus-infected cankers on living trees (Prospero et al. 2006), in chestnut stands with established hypovirulence the bark of freshly dead chestnut wood (e.g., broken branches, stumps) may represent an important source of hypovirulent inoculum, as experimentally shown by Meyer et al. (2019). The high canker infection rate by CHV1 could also result from a high efficiency of CHV1-infected *C. parasitica* conidia in transmitting the virus to other fungal strains following germination and physical contact. In most cankers we could only identify a single CHV1 strain which persisted over time, thus typically only one virus strain infected a canker. This supports previous findings that CHV1 infections appear to be caused by one infection event with the viral population within a canker originating from a single CHV1 strain, meaning infections are chronic for the fungal host (Leigh et al. 2021). Hence, contrary to what we observed for *C. parasitica* (see below), cankers appear to be a stable environment for CHV1. Second, the occurrence of effective vectors for CHV1. Conidia are mainly splash dispersed by rain or washed down the stem (Griffin 1986). However, birds, insects, mites or windborne dust may also transport them over longer distances (Heald and Studhalter 1914; Wendt et al. 1983; Russin et al. 1984). Third, as suggested by previous studies (e.g., Carbone et al. 2004; Brusini et al. 2011; Bryner and Rigling 2012), *in natura* the vegetative incompatibility system in *C. parasitica* seems to be more permeable than generally assumed from *in vitro* data. This becomes particularly evident when considering the factor time and the perennial nature of chestnut blight cankers. While in the first year of the experiment we observed virus transmission rates either lower than or within estimated values for several inoculated cankers, by the end of the experiment all cankers, regardless of the initially inoculated *C. parasitica* genotype, showed higher than expected virus infection rates. The observed differences among sites in the prevalence of CHV1 in the artificially initiated cankers of all *vc* types may be due to differences in any one of these three conditions mentioned above. However, other site-specific variables (e.g., air temperature and humidity) or system specific factors (e.g., local virus virulence) may have impacted CHV1 spread as well. Noteworthy, the lowest CHV1-prevalence at the end of the experiment was observed in Smolare, a North Macedonian population composed of a single *vc* type (EU-12). Given the lack of barriers due to vegetative incompatibility, in theory such clonal populations of *C. parasitica* should be easily colonized by CHV1. However, this population had also the lowest natural virus prevalence before the start of our experiment (Ježić et al. 2021), which likely impacted infection rate and drove the lower prevalence at the end of the experiment.

A CHV1 infection resulted in a significant decrease in expansion rate of bark cankers in Switzerland and North Macedonia, which was noticeable already after a relatively short time. In Croatia, at the end of the experiment still no remarkable differences in canker size were observed between virus-free and virus-infected cankers. This could be due to several reasons, including a rather late canker infection by CHV1 (especially in Ozalj), a lower virulence of local CHV1 strains, or a higher susceptibility of local chestnut populations to *C. parasitica* (Ježić et al. 2024). Due to virus infection, sporulation rate significantly decreased at all sites, though this effect was only visible by the end of the experiment. Reductions in expansion rates of virus-infected cankers have also previously been reported, e.g. in Bissegger et al. (1997) and Bryner et al. (2014). These results suggest that the effect of a CHV1 infection on canker development is mostly pronounced and fast. Keeping in mind that virus-free cankers expand quite rapidly (in our study: mean canker growth of 16 cm per year; data not shown), a quick response, i.e., stagnation of the canker growth after infection with CHV1, is likely needed for tree survival. Combining this finding with the efficient CHV1 spread, we expect the infected cankers not to be lethal for the chestnut sprouts. In several cases, we observed a callusing of hypovirus-infected cankers by the end of the experiment, which shows that the healing process induced by the host trees has already begun (see Suppl. material 4).

Since CHV1 infection seems to slow down but not entirely stop canker expansion, it is of the upmost importance that the infection of the canker with CHV1 takes place as early as possible after the initiation of a canker. The virus-induced reduction in *C. parasitica* sporulation on the surface of the bark cankers has an ambivalent effect for the biocontrol of chestnut blight since it lowers chances to spread for both the pathogen and the biocontrol agent. Given the importance of asexual spores for the spread of CHV1 (Prospero et al. 2006), an ideal CHV1 strain for biocontrol should be able to significantly reduce canker expansion without completely inhibiting the sporulation of the infected fungal strain, preferably producing conidia with a high CHV1 incidence.

Using microsatellite markers, new *C. parasitica* genotypes could be detected in the artificially initiated cankers of all *vc* types in all study sites revealing that perennial and apparently stable bark cankers are actually a highly dynamic environment for the pathogen. An existing canker may be invaded not only by new strains of its own *vc* type, but also by new strains of different *vc* types. A turnover of *C. parasitica* strains of different *vc* types was previously reported by Ježić et al. (2018) in natural bark cankers in Croatia that were re-sampled over a period of three years. Regarding epidemiology of chestnut blight this phenomenon shows that not only healthy bark, but also already colonized bark (canker) may be a suitable substrate for new *C. parasitica* infections.

Overall, the presence of the biological control agent in a bark canker did not seem to substantially affect chances of establishment of new fungal genotypes within a canker. On the contrary, the observed immigration of new *C. parasitica* genotypes into existing cankers provides the basis for the infection of these cankers by CHV1, as the virus has no extracellular phase and can spread only together with the fungus. Since CHV1 is only transmitted into asexual spores (conidia), we can assume that virus-infected conidia are carrying the virus to new cankers. Virus transmission then occurs after germination of the conidia followed by hyphal anastomosis with the *C. parasitica* strain causing the canker. Sexual ascospores (which are always virus-free) and virus-free conidia are not relevant for the spread of CHV1, but very likely also contributed to the establishment of new fungal strains within existing cankers, as new fungal genotypes were also found in virus-free cankers. Our study suggests that the immigration of new genotypes into existing cankers is an inherent process in the epidemiology of *C. parasitica*, which significantly contributes to the spread of CHV1.

## Conclusion

Our field experiment showed a high efficiency of CHV1 as biocontrol agent against chestnut blight in *C. parasitica* populations with naturally established hypovirulence. New cankers of different *vc* types became rapidly infected by CHV1, which mostly negatively impacted canker activity (i.e., growth and sporulation), increasing survival chances for the chestnut sprouts. Moreover, vegetative incompatibility barriers proved to be much less effective under field conditions than would be expected based on *in vitro* virus transmission experiments. This result is particularly favourable for the success of hypovirulence mediated biocontrol of chestnut blight in Europe although a potential critical point needs to be mentioned. The *vc* types of the *C. parasitica* genotypes used to initiate the cankers were already present in the specific chestnut stands or region, i.e., with the new cankers we did not significantly alter the genetic diversity (including *vc* type diversity) of the resident fungal population. Consequently, our results and conclusions cannot be automatically translated to genetically distant *vc* types that occur in *C. parasitica* populations outside Europe (e.g., Asia, Caucasian Georgia; Dutech et al. 2012; Prospero et al. 2013). Thus, to ensure success of hypovirulence in Europe also in the future, it is highly recommended to further implement current phytosanitary measures aiming at preventing the introduction of new *C. parasitica* genotypes which, regardless of their geographic origin, have been shown to be virulent on chestnut seedlings (Dennert et al. 2019).

## Additional information

### Use of AI

No use of AI was reported.

### Adherence to national and international regulations

All the fungal strains used in this study have been legally obtained, respecting the Convention on Biological Diversity (Rio Convention).

### Author ORCIDs

Simone Prospero https://orcid.org/0000-0002-9129-8556

Janine Melanie Schwarz https://orcid.org/0000-0003-2150-8156

Marin Ježić https://orcid.org/0000-0003-3029-7274

Deborah Marie Leigh https://orcid.org/0000-0003-3902-2568

Mirna Ćurković-Perica https://orcid.org/0000-0001-6592-6101

Marilena Idžojtić https://orcid.org/0000-0001-9728-3059

Zorana Katanić https://orcid.org/0000-0001-5555-6434

Ljiljana Krstin https://orcid.org/0000-0002-8542-2741

Lucija Nuskern https://orcid.org/0000-0002-2074-5339

Ivana Pavičić https://orcid.org/0009-0002-0600-9316

Igor Poljak https://orcid.org/0000-0002-8346-1683

Kiril Sotirovski https://orcid.org/0000-0002-6217-4706

Mihajlo Risteski https://orcid.org/0000-0002-3372-4991

Rosemary Vuković https://orcid.org/0000-0002-2324-3617

Daniel Rigling https://orcid.org/0000-0002-4338-5364

### Data availability

All of the data that support the findings of this study are available in the main text or Supplementary Information.

## Supplementary materials

Supplementary material 1 Resident and inoculated *Cryphonectria parasitica* multilocus genotypes in the six study sites Data type xlsx Explanation note Resident and inoculated microsatellite genotypes of *Cryphonectria parasitica*. https://binary.pensoft.net/file/1519068This dataset is made available under the Open Database License (http://opendatacommons.org/licenses/odbl/1.0/). The Open Database License (ODbL) is a license agreement intended to allow users to freely share, modify, and use this Dataset while maintaining this same freedom for others, provided that the original source and author(s) are credited. Simone Prospero, Janine Melanie Schwarz, Marin Ježić, Deborah Marie Leigh, Mirna Ćurković-Perica, Marilena Idžojtić, Zorana Katanić, Ljiljana Krstin, Lucija Nuskern, Ivana Pavičić, Igor Poljak, Kiril Sotirovski, Mihajlo Risteski, Rosemary Vuković, Daniel Rigling

Supplementary material 2 CHV1 strains detected in individual *Cryphonectria parasitica* bark cankers on at least two sampling dates in Switzerland, Croatia and North Macedonia Data type xlsx Explanation note CHV1 strains detected in the artificially initiated cankers. https://binary.pensoft.net/file/1519069This dataset is made available under the Open Database License (http://opendatacommons.org/licenses/odbl/1.0/). The Open Database License (ODbL) is a license agreement intended to allow users to freely share, modify, and use this Dataset while maintaining this same freedom for others, provided that the original source and author(s) are credited. Simone Prospero, Janine Melanie Schwarz, Marin Ježić, Deborah Marie Leigh, Mirna Ćurković-Perica, Marilena Idžojtić, Zorana Katanić, Ljiljana Krstin, Lucija Nuskern, Ivana Pavičić, Igor Poljak, Kiril Sotirovski, Mihajlo Risteski, Rosemary Vuković, Daniel Rigling

Supplementary material 3 Expected canker infection rate by CHV1 and observed cankers infection rates at the four sampling points at the six study sites in Switzerland, Croatia and North Macedonia Data type xlsx Explanation note Infection rates by CHV1 of the artificially initiated cankers. https://binary.pensoft.net/file/1519070This dataset is made available under the Open Database License (http://opendatacommons.org/licenses/odbl/1.0/). The Open Database License (ODbL) is a license agreement intended to allow users to freely share, modify, and use this Dataset while maintaining this same freedom for others, provided that the original source and author(s) are credited. Simone Prospero, Janine Melanie Schwarz, Marin Ježić, Deborah Marie Leigh, Mirna Ćurković-Perica, Marilena Idžojtić, Zorana Katanić, Ljiljana Krstin, Lucija Nuskern, Ivana Pavičić, Igor Poljak, Kiril Sotirovski, Mihajlo Risteski, Rosemary Vuković, Daniel Rigling

Supplementary material 4 Development of artificially initiated *Cryphonectria parasitica* bark cankers Data type pdf Explanation note Morphology of the artificially initiated bark cankers. https://binary.pensoft.net/file/1519071This dataset is made available under the Open Database License (http://opendatacommons.org/licenses/odbl/1.0/). The Open Database License (ODbL) is a license agreement intended to allow users to freely share, modify, and use this Dataset while maintaining this same freedom for others, provided that the original source and author(s) are credited. Simone Prospero, Janine Melanie Schwarz, Marin Ježić, Deborah Marie Leigh, Mirna Ćurković-Perica, Marilena Idžojtić, Zorana Katanić, Ljiljana Krstin, Lucija Nuskern, Ivana Pavičić, Igor Poljak, Kiril Sotirovski, Mihajlo Risteski, Rosemary Vuković, Daniel Rigling
