# Supplementary material for: Testing the efficiency of natural hypovirulence for biological control of chestnut blight under field conditions
Source: IMA Fungus. 2026 Jan 26;17:e173675. doi: 10.3897/imafungus.17.173675 (PMC12865391; doi:10.3897/imafungus.17.173675)
Supplement: Supplementary material 4 — Development of artificially initiated Cryphonectria parasitica bark cankers [file imafungus-17-e173675-s004.pdf]

(A) Canker Contone (CH) 15_2 (EU-12): Always CHV1-free

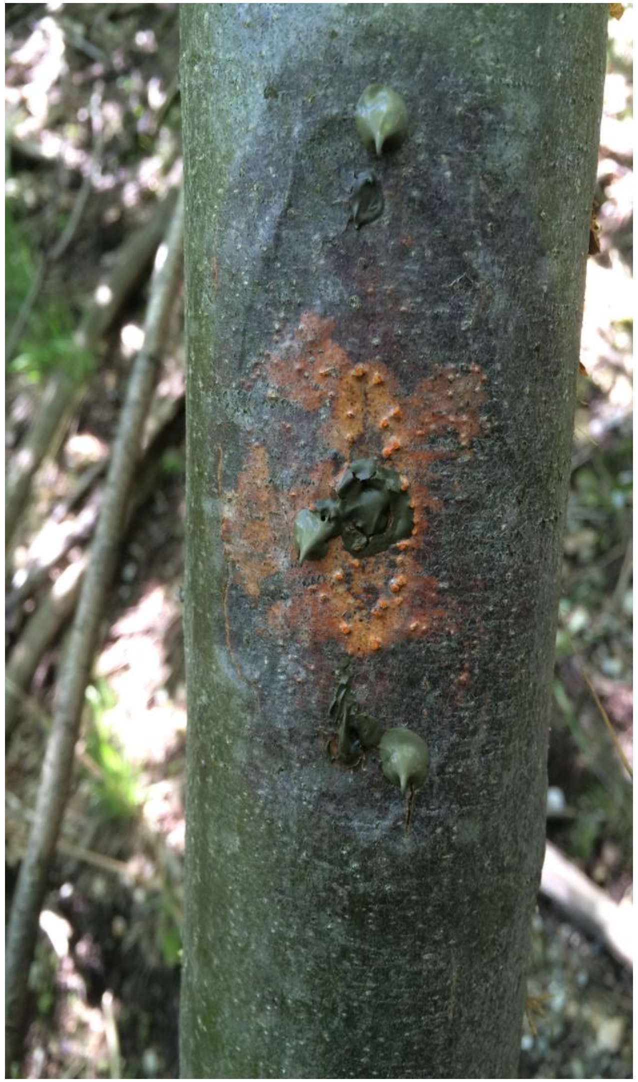

May 2015

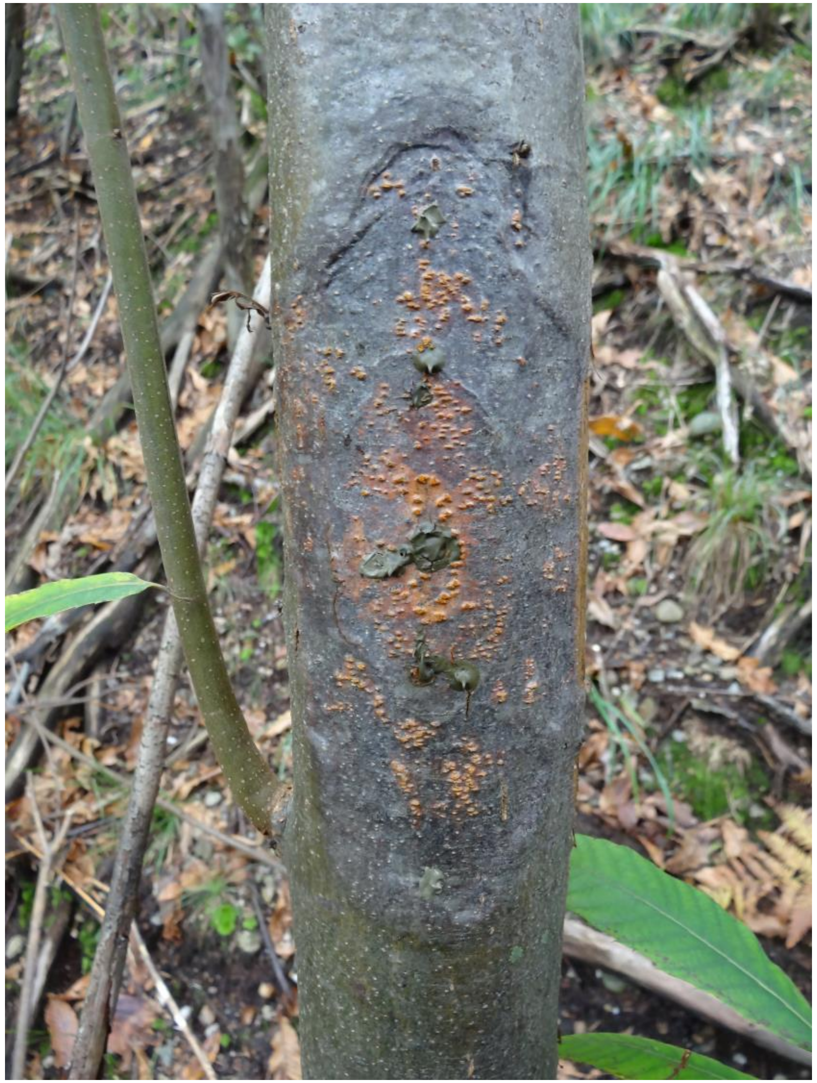

October 2015

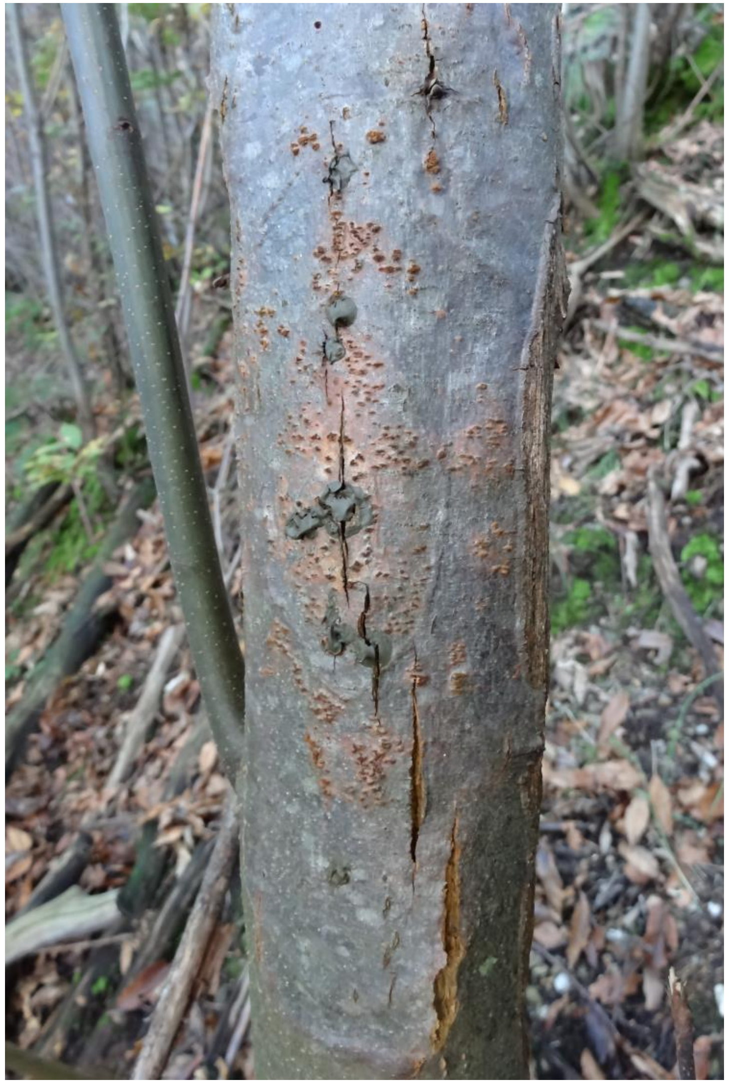

October 2016

(B) Canker Contone (CH) 6_1 (EU-1): CHV1-infected since October 2015 (not sampled before)

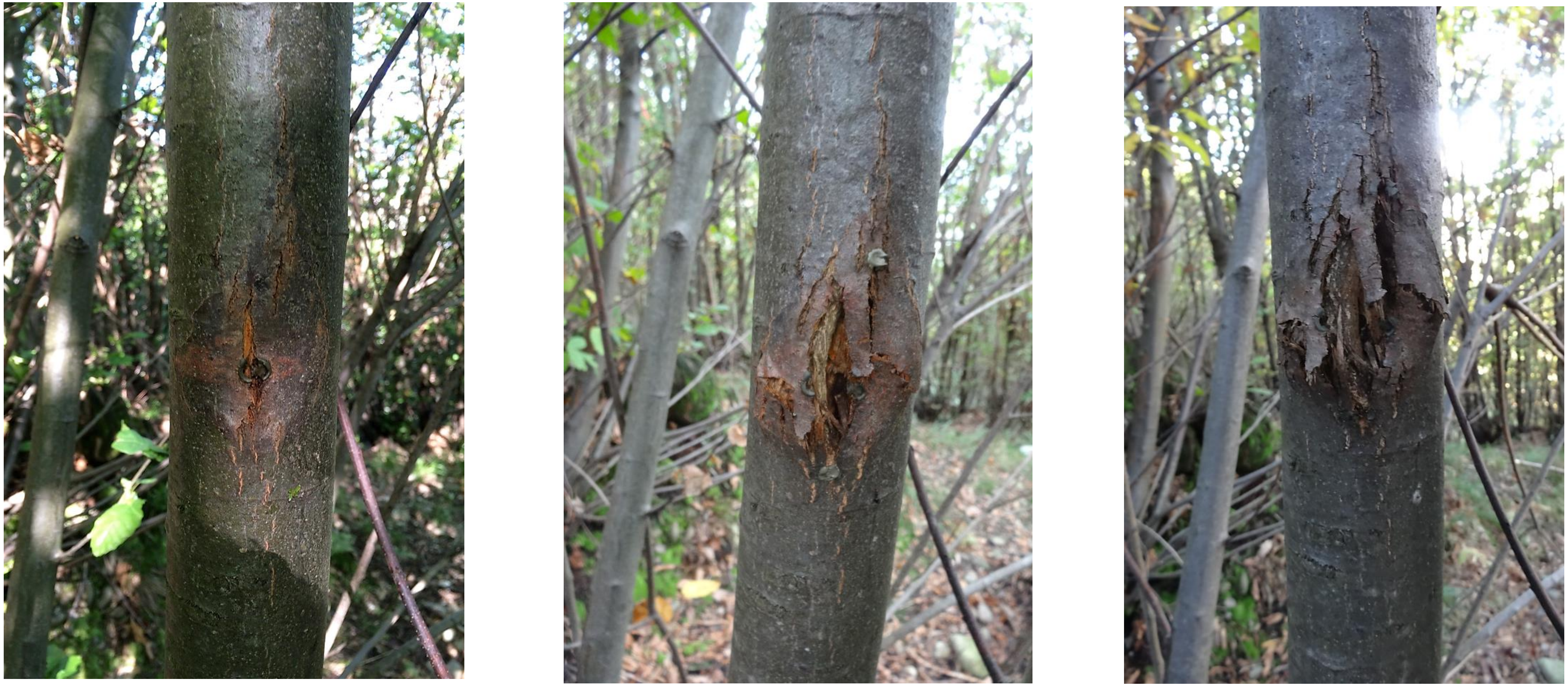

May 2015 | October 2015 | October 2016

Figure S1. Development of artificially initiated *Cryphonectria parasitica* bark cankers. (A) Canker that remained CHV1 free during the entire experiment; (B) Canker that became CHV1 infected in 2015.
